# Synthesis and Self-Assembled Behavior of pH-Responsive Chiral Liquid Crystal Amphiphilic Copolymers Based on Diosgenyl-Functionalized Aliphatic Polycarbonate

**DOI:** 10.3390/nano7070169

**Published:** 2017-07-04

**Authors:** Zhi-Hao Guo, Xiao-Feng Liu, Jian-She Hu, Li-Qun Yang, Zhang-Pei Chen

**Affiliations:** 1 Center for Molecular Science and Engineering, College of Science, Northeastern University, Shenyang 110819, China; 1610046@stu.neu.edu.cn (Z.-H.G.); 1510048@stu.neu.edu.cn (X.-F.L.); chenzhangpei@mail.neu.edu.cn (Z.-P.C.); 2 Key Laboratory of Reproductive Health and Medical Genetics, National Health and Family Planning Commission, Shenyang 110031, China; yanglq@lnszjk.com.cn

**Keywords:** diosgenin, liquid crystal, self-assembly, nanospheres, nanofibers

## Abstract

The morphological control of polymer micellar aggregates is an important issue in applications such as nanomedicine and material science. Stimuli responsive soft materials have attracted significant attention for their well-controlled morphologies. However, despite extensive studies, it is still a challenge to prepare nanoscale assemblies with responsive behaviors. Herein, a new chiral liquid crystal (LC) aliphatic polycarbonate with side chain bearing diosgenyl mesogen, named mPEG_43_-PMCC_25_-P(MCC-DHO)_15_, was synthesized through the ring-opening polymerization and coupling reaction. The self-assembled behavior of the LC copolymer was explored. In aqueous solution, the functionalized copolymer could self-organize into different nanostructures with changing pH value, such as nanospheres and nanofibers. This would offer new possibilities in the design of nanostructured organic materials.

## 1. Introduction

Self-assembled amphipathic polymers have wide applications in nanomedicine and material science. In mixed solvent and driven by hydrophobic chains, self-assembly of amphipathic macromolecular forms stable aggregates with hydrophobic chain as core and hydrophilic chain as shell. Recent research has indicated that the amphiphilic block copolymer reveals a controlled balance of amphiphilicity and can assume various well-organized architectures, such as spherical, rodlike, vesicles and lamellar structures, etc. [1,2,3,4,5,6,7]. In general, the morphologies and sizes of polymer micellar aggregates can be designed through molecular weight selection [8,9,10], chain architecture design [11,12,13], degree of crosslinking [6,14] and variation of solution conditions [1,15,16,17,18,19,20,21,22,23,24]. So, a variety of smart nanomaterial systems are constructed by association of various kinds of amphipathic polymers, including proteins, DNA, and phospholipids, through well-controlled self-assembly [25,26].

Chiral liquid crystal (LC) materials attracts considerable attention due to their excellent optical-electric properties, such as selective reflection of circular polarized light, highoptical rotatory power, circular dichroism, thermochromism, ferroelectricity and piezoelectricity [27]. In addition, chiral LC compounds are responsive to external stimuli, such as temperature, pressure, electromagnetic field, etc. Within this context and due to the unique properties, chiral steroidal compounds have become an important part of smart materials. Stupp et al. [28] first reported the synthesis of cholesterol end functionalized oligo(L-lactic acid) and its interaction with cells. Subsequently, Guo [29] and Cheng et al. [30] reported cholesteryl end-capped aliphatic polycarbonates and dicholesteryl end functionalized triblock poly(ε-caprolactone), respectively, as biodegradable polymers. Recently, the cholesteryl-functional polymers have been applied in the field of nanomedicine, such as drugs or nucleic acids delivery, complex hydrophobic therapeutics, etc. [31]. 

Diosgenin, an important kind of chiral steroidal compounds extracted from dioscorea zingiberensis, was used to synthesize sex hormones, steroids, and so on [32,33,34,35]. Some studies have revealed that diosgenin has multiple biological activities, such as anti-infection [36,37], anti-thrombus [38], enhancing immune activity of T-cells [39], and inhibition of cancer cell proliferation [40]. The diosgenin and its derivatives can be used as bioactive LC functional mesogens for their good biocompatibility. In addition, to the best of our knowledge, although biodegradable copolymers containing side active groups for the drug delivery has been studied [41,42], few research on the biodegradable aliphatic polycarbonate containing side diosgenyl groups as biomesogenic units is reported. Therefore, it’s an interesting research on synthesizing biodegradable side chain chiral LC polycarbonate derived from diosgenin, studying their self-assembled behavior and exploring the potential applications in the field of biomedical science, such as drug delivery and tissue engineering templates.

**Scheme 1:**
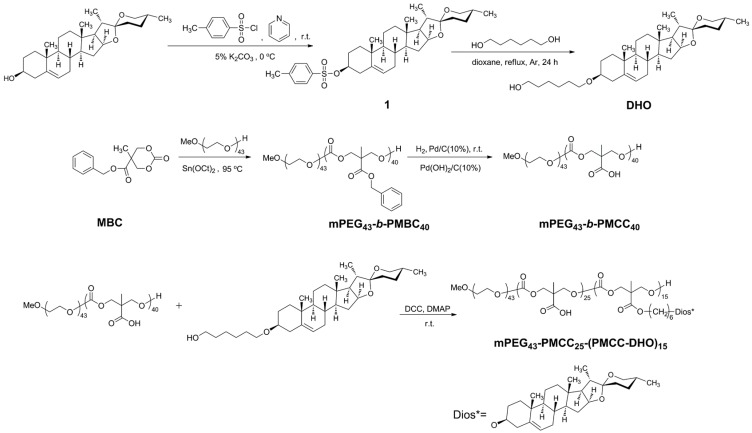
Synthetic route to copolymer.

Hence, on the basis of the above consideration, a new chiral LC polycarbonate with side chain bearing diosgenyl mesogen, named as mPEG_43_-PMCC_25_-P(MCC-DHO)_15_, is synthesized through the ring-opening polymerization and coupling reaction (Scheme 1). A side chain functionalized diosgenin with hydrophobic moiety is incorporated onto the aliphatic polycarbonate for the introduction of physicochemical functionality such as mesomorphism. Furthermore, due to the existence of the multifunctional diosgenyl, the properties of mPEG_43_-PMCC_25_-P(MCC-DHO)_15_ such as phase behavior and self-assembled behavior are studied.

## 2. Results and Discussion

### 2.1. Synthesis and Structural Characterization of mPEG_43_-b-PMBC_40_

For the preparation of aliphatic polycarbonates the ring-opening polymerization is an efficient method. In general, the ring-opening polymerization of aliphatic cyclic carbonate monomer could be carried out in the presence of an initiator with hydroxyl groups such as mPEG. In this study, 5-methyl-5-benzyloxycarbonyl-1, 3-dioxan-2-one (MBC) was first obtained by a previously reported procedure with a slight modification [43], proton nuclear magnetic resonance (^1^H NMR) spectra of MBC is shown as Figure S1. Then mPEG_43_-*b*-PMBC_40_ was synthesized through the ring-opening polymerization of the functionalized cyclic carbonate monomer MBC using Sn(Oct)_2_ as a catalyst in the presence of mPEG_43_ as a macroinitiator. The ring-opening polymerization was confirmed by ^1^H NMR spectra (Figure S2), Fourier transform infrared (FT-IR) spectroscopy (Figure S3) and gel permeation chromatographic (GPC) (Figure S4) analysis. After the ring-opening polymerization, the corresponding signals of methylene proton in the cyclic monomer MBC at 4.69 and 4.19 ppm shifted to 4.29 ppm. Compared with the C=O stretching vibration bands of MBC at 1752 and 1737 cm^−1^, the *ν*(C=O) of mPEG_43_-*b*-PMBC_40_ only appeared a peak at 1754 cm^−1^. The GPC analysis confirmed the degree of polymerization and dispersity of the aliphatic polycarbonates, and indicated that the monomer reacted completely.

### 2.2. Synthesis and Structural Characterization of mPEG_43_-b-PMCC_40_

The preparation of mPEG_43_-*b*-PMCC_40_ was carried out by construction of a polycarbonate containing one reactive side chain carboxylic acid functionality at each repeat unit. mPEG_43_-*b*-PMCC_40_ was obtained by hydrogenolysis reaction to result in the deprotection of side benzyl groups for mPEG_43_-*b*-PBMC_40_ using H_2_ gas, Pd/C and Pd(OH)_2_/C as co-catalysts. The cleavage of the benzyl ester groups was confirmed by ^1^H NMR (Figure S5) and FT-IR (Figure S3) spectroscopy. Compared with the ^1^H NMR of mPEG_43_-*b*-PBMC_40_, the proton signals of the benzyl groups at 7.35–7.28 and 5.14 ppm disappeared for mPEG_43_-*b*-PMCC_40_, in addition, the FT-IR spectroscopy of mPEG_43_-*b*-PMCC_40_ appeared typical peak of carboxyl group at 3600–2500 cm^−1^, which indicated that the elimination of the benzyl groups was complete.

### 2.3. Synthesis and Structural Characterization of mPEG_43_-PMCC_25_-P(MCC-DHO)_15_

**Figure 1:**
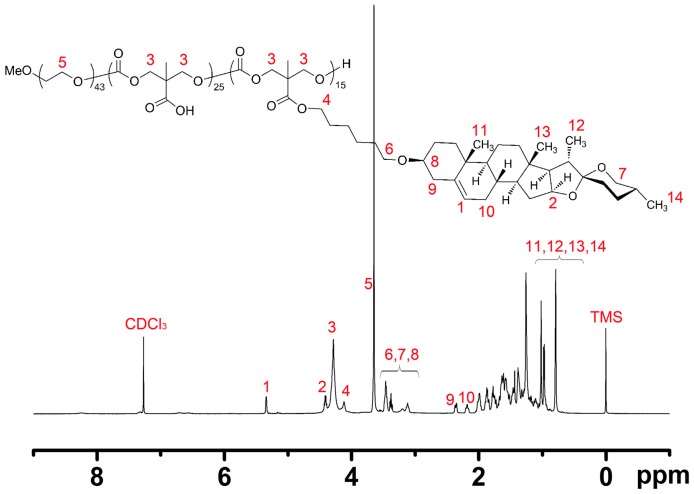
^1^H NMR spectra of mPEG_43_-PMCC_25_-P(MCC-DHO)_15_.

The availability of pendant diosgenyl groups for further functionalization was demonstrated by an esterification reaction between mPEG_43_-*b*-PMCC_40_ and 6-diosgenoxyhexane-1-ol (DHO) using DCC as a coupling agent and DMAP as a catalyst. The formation of ester bonds was confirmed by a shift of the methane (HOC***H***_2_–) peak from 3.65 ppm in DHO to 4.12 ppm in mPEG_43_-PMCC_25_-P(MCC-DHO)_15_, the number of DHO was determined by the integration values ratio of the proton signals at 4.12 for O=COC***H***_2_– to 3.65 for C***H***_2_ in mPEG_43_. Figure 1 shows the ^1^H NMR spectra of mPEG_43_-PMCC_25_-P(MCC-DHO)_15_. In addition, the *ν*(–COOH) at 3600–2500 cm^−1^ for mPEG_43_-*b*-PMCC_40_ were not obvious, these results showed that the diosgenyl groups were successfully introduced to side chain of the aliphatic polycarbonates. 

### 2.4. Liquid Crystal Behaviour of mPEG_43_-PMCC_25_-P(MCC-DHO)_15_

**Figure 2:**
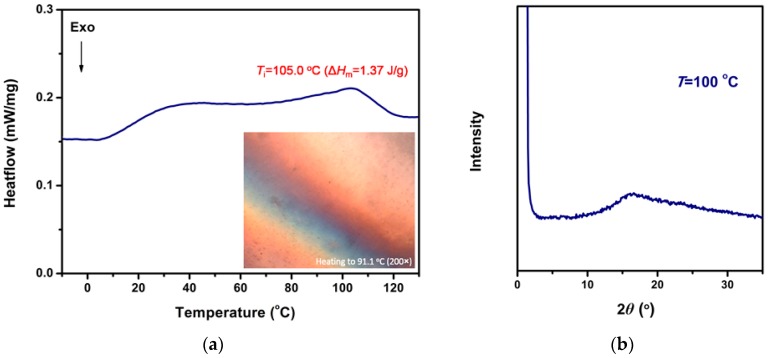
(**a**) Differential scanning calorimetry (DSC) curves of mPEG_43_-PMCC_25_-P(MCC-DHO)_15_, the inset at lower right is photograph on heating to 91.1 °C recorded by polarizing optical microscopy (POM); (**b**) X-ray diffraction (XRD) curve of mPEG_43_-PMCC_25_-P(MCC-DHO)_15_ at 100 °C.

From a scientific and commercial point of view, chiral LC materials with helical supramolecular structure are fascinating. As known, chirality can be introduced into LC molecules at various levels, and is usually located in the terminal position of the mesogenic core. The rod-like, chiral molecules responsible for the macroscopical alignment of the mesogenic domains can produce cholesteric or a chiral smectic phase. The LC behaviour of mPEG_43_-PMCC_25_-P(MCC-DHO)_15_ was investigated with differential scanning calorimetry (DSC), X-ray diffraction (XRD) and polarizing optical microscopy (POM). According to DSC heating curve, a typical glass transition appeared indicating that the LC copolymer was amorphous polymer (Shown in Figure 2a). In addition, mPEG_43_-PMCC_25_-P(MCC-DHO)_15_ exhibited a mesomorphism to isotropic phase transition at 105.0 °C because the mesogenic DHO units were introduced to side-chain of the polycarbonate. POM observations showed that mPEG_43_-PMCC_25_-P(MCC-DHO)_15_ exhibited obvious mesomorphism at body temperature (≈37 °C). When the temperature increased to 116.9 °C, the birefringence disappeared. To further identify mesophase structure, variable-temperature XRD studies were carried out. Figure 2b shows the XRD patterns of mPEG_43_-PMCC_25_-P(MCC-DHO)_15_ at 100 °C, only a weak and diffuse peak at wide angle was observed, indicating the formation of a cholesteric phase.

### 2.5. pH Responsive Self-Assembly of mPEG_43_-PMCC_25_-P(MCC-DHO)_15_

**Figure 3:**
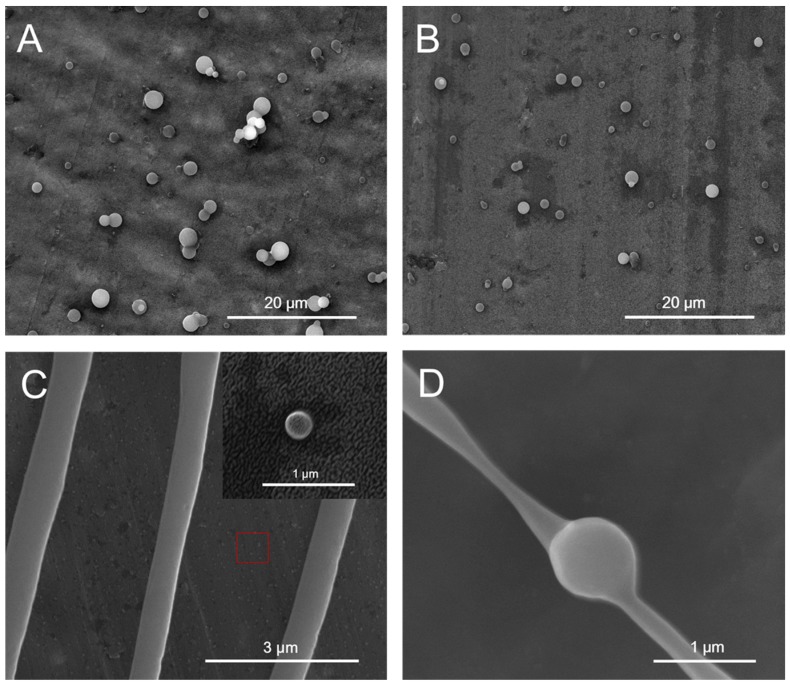
SEM images of mPEG_43_-PMCC_25_-P(MCC-DHO)_15_ self-assembly obtained in water at (**A**) pH = 4.0, (**B**) pH = 6.0, (**C**) pH = 7.0 and (**D**) pH = 8.0. The inset at higher right in (**C**) is an enlargement of the spherical aggregates in the selected rectangular area.

The morphologies of mPEG_43_-PMCC_25_-P(MCC-DHO)_15_ nano-aggregates were analyzed by scanning electron microscopes (SEM) and transmission electron microscopy (TEM). SEM images of the aggregates are shown in Figure 3. An interesting phenomenon was that the micellar aggregates in water could show different pH-responsive morphologies including spherical and nanofibers aggregates. In acidic conditions (pH < 7.0), the morphology of aggregates exhibited nanospheres (Figure 3A,B), however, when the pH increased to 7.0, the nanospheres began to stretch to nanofibers, and a few spherical structures (average radius of spheres: 115.1 nm) were still visible (Figure 3C). Fortunately, the clear transition process of nanospheres to nanofibers was observed at pH = 8.0 (Figure 3D). To further confirm the existence of the morphologies in mPEG_43_-PMCC_25_-P(MCC-DHO)_15_ sample, it was subjected to a transmission electron microscope (TEM) (Figure S6), the images given a significant evidence that the micellar aggregates were solid rather than hollow. When pH < 7.0, the nanospheres could merge with others, as shown in the Figure 3A,B, this phenomenon led that the sizes of the most aggregates were larger than the practical sizes, therefore, the minimum size of the spherical aggregates recorded by electron microscopes was the nearest to their actual size. Similarly, the nanofibers could also merge into others and large diameter nanofibers formed.

**Figure 4:**
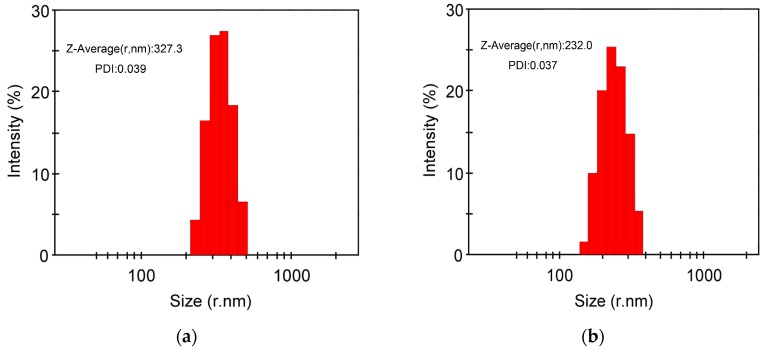

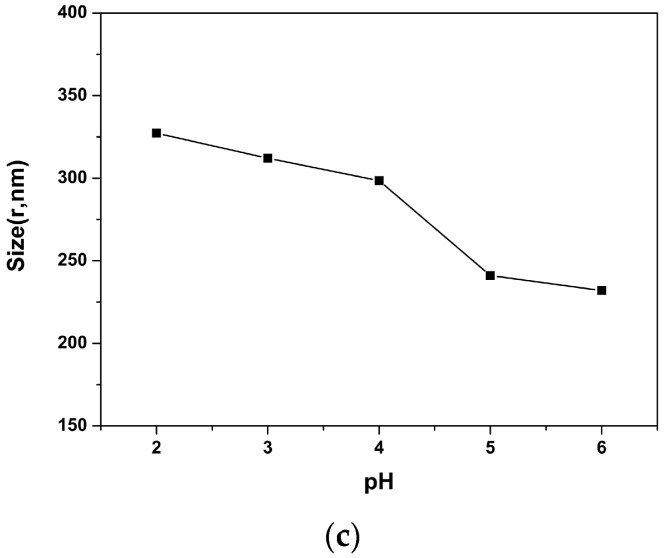
Dynamic light scattering (DLS) histogram of mPEG_43_-PMCC_25_-P(MCC-DHO)_15_ in water at (**a**) pH = 2.0 and (**b**) pH = 6.0, (**c**) size of mPEG_43_-PMCC_25_-P(MCC-DHO)_15_ in various pH at 25 °C.

To trace structural changes in the spherical assemblies, the sizes of the aggregates were also measured by DLS with changing pH value. DLS histograms of mPEG_43_-PMCC_25_-P(MCC-DHO)_15_ at pH = 2.0 and 6.0 are shown as Figure 4a,b, and the effect of pH value on the sizes of aggregates is shown as Figure 4c. When pH = 2.0, average radius of the spherical assemblies was 327.3 nm (PDI = 0.039), with increasing pH value to 6.0, the radius of nanospheres decreased to 232.0 nm (PDI = 0.037). This interesting change provides significant evidence that the spherical assemblies were in response to pH.

**Figure 5:**
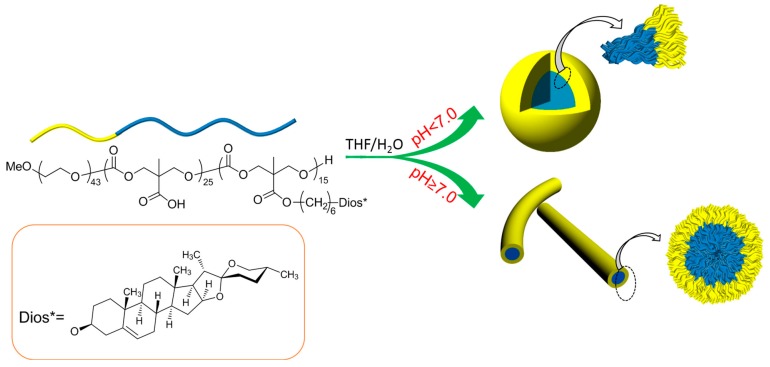
Self-assembly of mPEG_43_-PMCC_25_-P(MCC-DHO)_15_ with nanospheres and nanofibers micelles.

As known, LC materials have good self-organization orientational behavior [44,45,46]. The ability of side diosgenyl groups to form mesophase will be anticipated to provide an ordered structure for the intermolecular self-assembly. With the incorporation of the mesogen, the movement and arrangement of the amphiphilic aliphatic polycarbonates were influenced by ordered mesophase. In tetrahydrofuran (THF), the macromolecular chain of mPEG_43_-PMCC_25_-P(MCC-DHO)_15_ completely stretched, with dialyzing against selective solvent water, the side diosgenyl groups rearranged and formed ordered mesophase structure, and the hydrophobic segments were frozen in the meanwhile, then, the macromolecular chain collapsed, and the core-shell structure was formed with hydrophobic chain as core and hydrophilic chain as shell (Figure 5). In acidic conditions (pH < 7.0), the nanospheres merged with others and stable aggregates were formed. However, as increasing pH value, the carboxyl groups of mPEG_43_-PMCC_25_-P(MCC-DHO)_15_ tend to ionize to stabilize the aggregates [47], which made the merging process difficult due to the electrostatic repulsions, and the decreasing radius of nanospheres. When pH ≥ 7.0, the nanospheres were highly charged and the strong repulsive electrostatic interactions provided a driving force for stretching the core-shell structure, which in turn influenced the chain conformation from compact nanospheres at low pH to extended nanofibers at high pH.

## 3. Materials and Methods

Tosyl chloride, palladium 10% on carbon, palladium hydroxide, and stannous octoate were purchased from Sinopharm Chemical Reagent Co., Ltd. (Shenyang, China). Diosgenin was purchased from Wuhan Chemical Industry Co., Ltd. (Wuhan, China). 1,6-Hexanediol was purchased from Qingdao Lilai Fine Chemical Industry Co., Ltd. (Qingdao, China). 5-methyl-5-benzyloxycarbonyl-1,3-dioxan-2-one (MBC) were synthesized as reported [43]. All solvents and reagents used in this study were purified with standard methods.

FT-IR spectra were recorded as a KBr disk on a PerkinElmer spectrum One (B) spectrometer (PerkinElmer, Foster City, CA, USA). ^1^H NMR spectra were measured using a Bruker ARX 600 (Bruker, Karlsruhe, Germany) high-resolution NMR spectrometer, and chemical shifts were reported in parts per million with tetramethylsilane (TMS) as an internal standard. Average molecular weights of the polymers were characterized with gel permeation chromatographic (GPC) measurements at room temperature on a Waters 1515 instrument (Waters, Milford, MA, USA) calibrated with a polystyrene standard, and using THF as an eluent. The thermal behavior was determined with a Netzsch 204 (Netzsch, Hanau, Germany) differential scanning calorimetry (DSC) equipped with a cooling system at a heating and cooling rate of 10 °C/min in a nitrogen atmosphere. The mesomorphism and phase transition temperature were observed with a Leica DMRX (Leica, Wetzlar, Germany) polarizing optical microscopy (POM) equipped with a Linkam THMSE-600 (Linkam, London, UK) cool and hot stage. X-ray diffraction (XRD) measurements were performed with a nick-filtered Cu-K_α_ radiation with a Bruker D8 Advance (Bruker, Karlsruhe, Germany) power diffractometer. Dynamic light scattering (DLS) measurements were carried out using a Zetasizer Nano ZS/ZEN3690 (Malvern, England) for the analysis of block copolymer aggregates hydrodynamic radii in a dilute solution. A backscattering measurement angle of 173° was used, repeating the measurement three times for each sample, with 12–15 runs per measurement. Scanning electron microscope (SEM) measurements were performed on an Inspect F50 (FEI., Hillsboro, OR, USA). The samples for SEM observations were prepared by depositing several drops of the samples suspension onto the surface of cleaned aluminum foil, and the samples were quenched by liquid nitrogen, then freeze-dried in vacuum at −50 °C for 24 h. The samples were coated with a thin film of gold before measuring. For transmission electron microscopy (TEM), the samples suspension was deposited onto a carbon-coated copper TEM grid and flash-frozen in liquid nitrogen, then freeze-dried in vacuum at −50 °C for 24 h. The frozen sample was scanned at 120 kV (JEM-2100, JEOL Ltd., Tokyo, Japan), the size of the particles was measured manually.

Synthesis of DHO. The compound **1** was obtained by reacting diosgenin and tosyl chloride using pyridine as solvent. Then, put compound **1** (14.2 g, 25 mmol), 1,6-hexanediol (59 g, 0.5 mol), and anhydrous 1,4-dioxane (300 mL) in a 500 mL three-neck flask with a magnetic stir bar. After the reaction mixture was refluxed for 24 h under an argon atmosphere, then concentrated under reduced pressure, the residue was dissolved in 100 mL of ethyl acetate. The resulting solution was washed three times with water to remove 1,6-hexanediol, the combined organic layer was dried over anhydrous magnesium sulfate, and then filtered. The filtrate was evaporated to dryness and the crude product was purified by silica gel column chromatography (petroleum ether/ethyl acetate = 3:1) and dried in a vacuum to result in a white powder. FT-IR (KBr, cm^−1^): 3368 (–OH); 2948, 2868 (CH_3_–, –CH_2_–); 1242, 1096 (C–O–C). ^1^H NMR (600 MHz, CDCl_3_, δ, ppm): 5.35 (d, 1H, *J* = 6.0Hz, –C***H***=C<), 4.41 (q, 1H, *J* = 7.8Hz, <C***H***O–), 3.65 (t, 2H, *J* = 6.6Hz, HOC***H***_2_–), 3.49–3.38 [m, 4H, HO(CH_2_)_5_C***H***_2_O– and –OC***H***_2_CH<], 3.12 [m, 1H, HO(CH_2_)_6_OC***H***<], 2.37–0.79 [m, 45H, ***H***-diosgenyl and–(C***H***_2_)_4_–]. ^13^C NMR (600 MHz, CDCl_3_, δ, ppm): 140.67, 121.20, 109.15, 80.70, 79.10, 71.38, 67.85, 66.70, 62.09, 56.41, 49.97, 46.45, 41.48, 40.14, 39.67, 38.88, 37.13, 37.03, 31.96, 31.72, 31.46, 31.29, 30.29, 30.02, 28.78, 28.54, 28.41, 25.82, 20.74, 19.30, 17.05, 16.18, 14.43.

Synthesis of mPEG_43_-*b*-PMBC_40_. MBC (20.0 g, 0.08 mol), mPEG_43_ (3.8 g, 2.0 mmol), and Sn(Oct)_2_ toluene solution (1.62 mL, 0.123 mol/L) were transferred into a glass polymerization flask, then the flask was sealed under vacuum. After the mixture was reacted for 24 h at 95 °C in an oil bath, the polymer was precipitated in ice-cold methanol (400 mL). Then, the crude polymer was redissolved in dichloromethane and reprecipitated again in ice-cold methanol. The polymer was dried in a vacuum until a constant sample mass was obtained. *M*_n_^NMR^ = 11,900 g/mol; *M*_n_^GPC,THF^ = 9630 g/mol; PDI^THF^ = 1.62. FT-IR (KBr, cm^−1^): 2974, 2883 (CH_3_–, –CH_2_–); 1754 (C=O); 1498 (Ar–); 1246, 1140 (C–O–C). ^1^H NMR (600 MHz, CDCl_3_, δ, ppm): 7.35–7.28 (Ar–***H***), 5.14 (ArC***H***_2_O), 4.29 (C***H***_2_ in polycarbonate chain), 3.65 (C***H***_2_ in mPEG_43_), 1.24 (C***H***_3_ in polycarbonate chain). ^13^C NMR (600 MHz, CDCl_3_, δ, ppm): 171.83, 154.27, 135.30, 128.51, 128.16, 127.87, 70.49, 68.51, 67.08, 46.49, 17.43. The DP^NMR^ of mPEG_43_-*b*-PMBC = 40.

Synthesis of mPEG_43_-*b*-PMCC_40_. mPEG_43_-*b*-PMBC_40_ (20.0 g), 10% Pd/C (1.0 g), Pd(OH)_2_/C (1.0 g) and methanol/THF (300 mL, 1:1) were added to a 500-mL three neck flask with a magnetic stir bar under an argon atmosphere. The reaction mixture was stirred for 48 h at 35 °C under a hydrogen gas pressure system. The Pd/C and Pd(OH)_2_/C were filtered off, the filtrate was evaporated to dryness. The product was dried under vacuum until a constant sample mass was obtained. FT-IR (KBr, cm^−1^): 3600–2500 (–COOH); 1753 (C=O); 1251, 1145 (C–O–C). ^1^H NMR (600 MHz, CDCl_3_, δ, ppm): 4.19 (C***H***_2_ in polycarbonate chain), 3.50 (C***H***_2_ in mPEG_43_), 1.14 (C***H***_3_ in polycarbonate chain). ^13^C NMR (600 MHz, DMSO, δ, ppm): 173.91, 154.45, 70.16, 69.23, 46.02, 17.36.

Synthesis of mPEG_43_-PMCC_25_-P(MCC–DHO)_15_. In a 250-mL three-neck flask with magnetic stir bar, mPEG_43_-*b*-PMCC_40_ (4.98 g), *N*,*N*′-dicyclohexyl carbodiimide (DCC) (5.95 g), and *N*,*N*′-dimethylaminopyridine (DMAP) (0.60 g) were dissolved in 100 mL of THF. DHO (14.9 g, 28.9 mmol) dissolved in 50 mL of THF was added dropwise to the above mixture. The reaction mixture was stirred for 48 h at 30 °C. The resulting mixture was washed with deionized water, and frozen at −20 °C for 15 min, then a solid, *N*,*N′*-dicyclohexyl urea, was precipitated by high speed centrifuge and filtered off. The filtrate was dried with anhydrous MgSO_4_, filtered and evaporated to dryness. The crude product was purified by dissolving it in dichloromethane and precipitated in ice-cold methanol, and then dried in a vacuum until a constant sample mass was obtained. *M*_n_^NMR^ = 15,755 Da; *M*_n_^GPC,THF^ = 12,789 g/mol; PDI^THF^ = 1.69. FT-IR (KBr, cm^−1^): 2931, 2857 (CH_3_–, –CH_2_–), 1754 (C=O), 1248, 1143 (C–O–C). ^1^H NMR (600 MHz, CDCl_3_, δ, ppm): ^1^H NMR (δ, ppm from TMS in CDCl_3_): *δ* = 5.34 (t, –C***H***=C<), 4.40 (q, >C***H***O–), 4.29 (m, C***H***_2_ in polycarbonate chain), 4.12 (m, O=COC***H***_2_–), 3.65 (t, C***H***_2_ in mPEG_43_), 3.47–3.38 (m, –(CH_2_)_5_C***H***_2_O– and –OC***H***_2_CH<], 3.12 [m, –(CH_2_)_6_OC***H***<], 2.36 [ (m, OCH_2_C***H***_2_C(CH_2_)=CH– (*eq*) ], 2.19 [m, >C=CHC***H***_2_CH< (*eq*)], 1.23 (C***H***_3_ in polycarbonate chain), 1.02 [s, –HC=C(CH_2_)C(CH_2_)_2_C***H***_3_], 0.98 [d, –CH(CH<)C***H***_3_], 0.79 [m, –C(CH_2_–)(CH<)C***H***_3_ and OCH_2_CH(CH_2_)C***H***_3_], 2.03–1.30 (rest of protons from diosgenyl). ^13^C NMR (600 MHz, DMSO, δ, ppm): 172.50, 154.32, 141.05, 121.06, 109.21, 80.74, 78.83, 70.47, 68.49, 67.86, 66.75, 65.38, 62.01, 56.44, 52.45, 50.04, 46.39, 41.51, 40.18, 39.70, 39.08, 37.17, 36.96, 33.83, 32.63, 32.01, 31.76, 31.32, 30.21, 30.03, 29.61, 28.71, 25.96, 20.76, 19.32, 17.38, 17.05, 16.20, 14.44.

Self-assembly of mPEG_43_-PMCC_25_-P(MCC-DHO)_15_. The copolymer (5.0 mg) was dissolved in THF (5.0 mL), then the polymer solution in THF was transferred to a prewashed dialysis membrane with a molecular weight cutoff (MWCO) of 3500 Da (Spectra/Por) and was dialyzed at room temperature against water (1.5 L) with different pH (2.0–10.0), the pH controlled by HCl or ammonium hydroxide, and the water changed at 6, 12, and 24 h. Typically the final concentration of the polymer solution after dialysis was about 0.6 mg/mL.

## 4. Conclusions

In summary, a new amphipathic chiral LC polycarbonate containing side diosgenyl groups was successfully synthesized. The amphiphilic block copolymers mPEG_43_-PMCC_25_-P(MCC-DHO)_15_ could exhibit an LC state below body temperature and have unique self-assembly behaviors in aqueous solution, both in sphere micelles (pH < 7.0) and fiber-like micelles (pH ≥ 7.0). This is an important result for potential applications taking advantage of these different morphologies. In addition, the chiral LC polymers studied here are based on entirely biocompatible diosgenyl mesogens. Our target is using the system to design drug carriers for future applications. It remains a challenge to establish how to control the polymer morphology transition precisely at different pH values, which will be a main subject of further study.

## Supplementary Materials

The following are available online at http://www.mdpi.com/2079-4991/7/7/169/s1, Figure S1: ^1^H NMR spectra of MBC, Figure S2: ^1^H NMR spectra of mPEG_43_-*b*-PMBC_40_, Figure S3: FT-IR spectroscopy of copolymers, Figure S4: GPC profiles (THF as an eluent, 1 mL/min) of mPEG_43_-*b*-PMBC_40_, Figure S5: ^1^H NMR spectra of mPEG_43_-*b*-PMCC_40_, Figure S6: TEM images of mPEG_43_-PMCC_25_-P(MCC-DHO)_15_ self-assembly obtained in water at (A) pH = 6 and (B) pH = 8.
