# Supplementary material for: Synthesis and Self-Assembled Behavior of pH-Responsive Chiral Liquid Crystal Amphiphilic Copolymers Based on Diosgenyl-Functionalized Aliphatic Polycarbonate
Source: Nanomaterials (Basel). 2017 Jul 4;7(7):169. doi: 10.3390/nano7070169 (PMC5535235; doi:10.3390/nano7070169)
Supplement: Supplementary file 1 [file nanomaterials-07-00169-s001.pdf]

Supporting Information for

Synthesis and Self-Assembled Behaviour of pH-Responsive Liquid Crystal Amphiphilic Copolymers Based on Diosgenyl-Functionalized Alphatic Polycarbonate

Zhi-Hao Guo [1], Xiao-Feng Liu [1], Jian-She Hu [1,*], Li-Qun Yang [2,*] and Zhang-Pei Chen [1]

[1] Center for Molecular Science and Engineering, College of Science, Northeastern University, Shenyang 110819, China; 1610046@stu.neu.edu.cn (Z.-H.G.); 1510048@stu.neu.edu.cn (X.-F.L.); chenzhangpei@mail.neu.edu.cn (Z.-P.C)

[2] Key Laboratory of Reproductive Health and Medical Genetics, National Health and Family Planning Commission, Shenyang, 110031, China; yanglq@lnszjk.com.cn (L.-Q.Y)

* Correspondence: E-mail: hujs@mail.neu.edu.cn (J.-S.H.); Tel.: +86-024-8368-7671

MBC ^1H NMR (600 MHz, $CDCl_3$, δ, ppm): 7.40-7.33 (m, 5H, J=7.0, ***H***-1), 5.22 (s, 2H, ***H***-2), 4.71 (d, 2H, J=10.8, ***H***-3), 4.21 (d, 2H, J=10.8, ***H***-4), 1.33 (s, 3H, ***H***-5)。

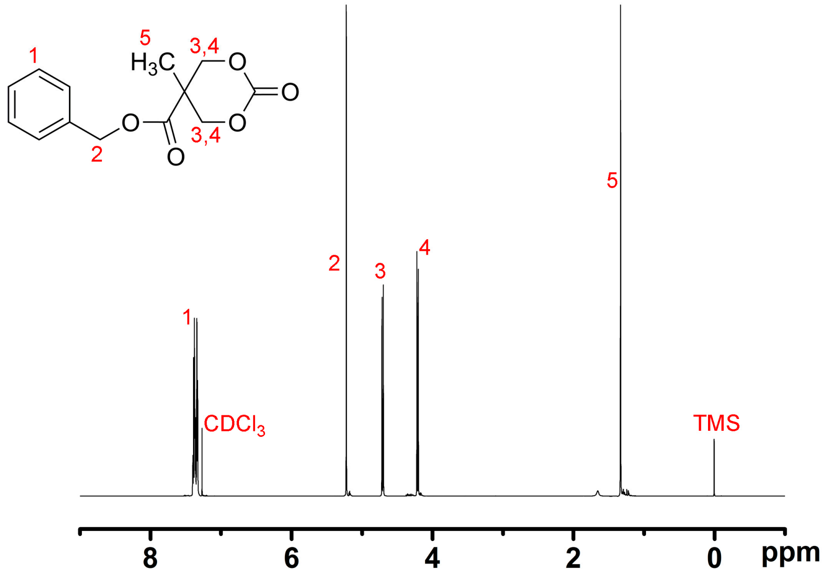

Figure S1. ^1H NMR spectra of MBC.

mPEG$_{43}$-*b*-PMBC$_{40}$ ^{1}H NMR (600 MHz, $CDCl_3$, δ, ppm): 7.35-7.28 (m, ***H***-1), 5.14 (s, ***H***-2), 4.29 (m, ***H***-3), 3.65 (s, ***H***-4), 1.24 (s, ***H***-5)。

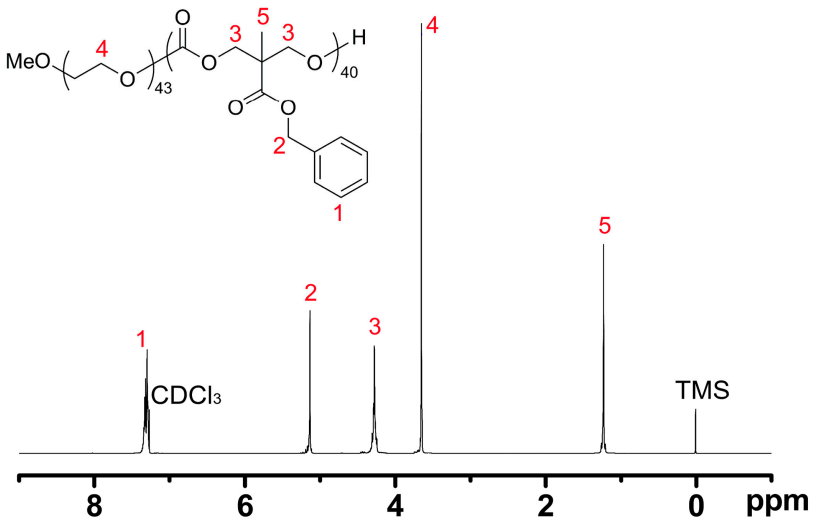

Figure S2. ^{1}H NMR spectra of mPEG$_{43}$-*b*-PMBC$_{40}$.

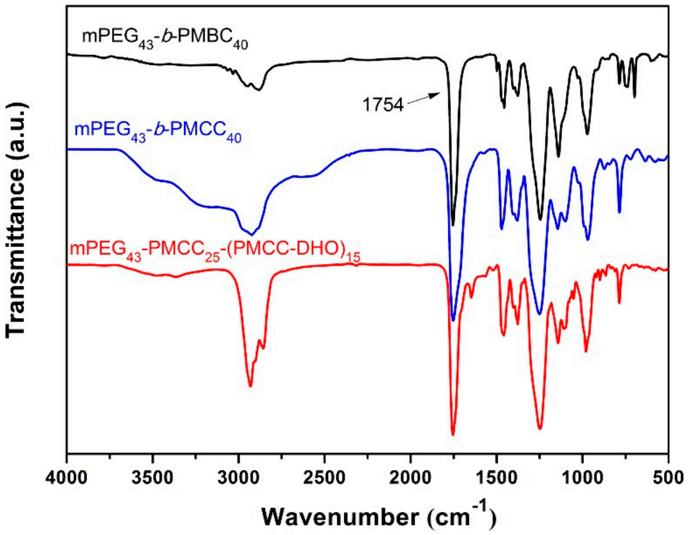

Figure S3. FT-IR spectroscopy of copolymers.

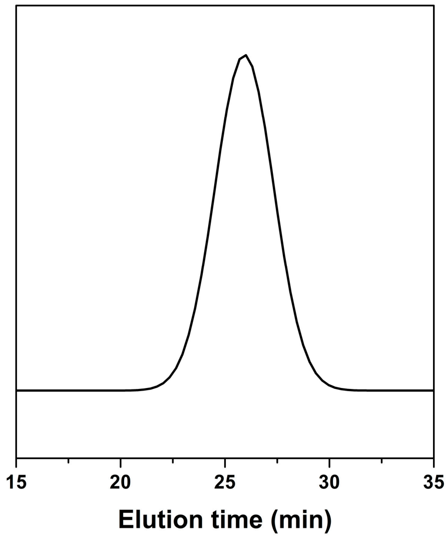

Figure S4. GPC profiles (THF as an eluent, 1 mL/min) of mPEG$_{43}$-*b*-PMBC$_{40}$.

mPEG$_{43}$-*b*-PMCC$_{40}$ ^{1}H NMR (600 MHz, $CDCl_3$, δ, ppm): 4.19 (m, ***H***-1), 3.50 (s, ***H***-2), 1.14 (s, ***H***-3)。

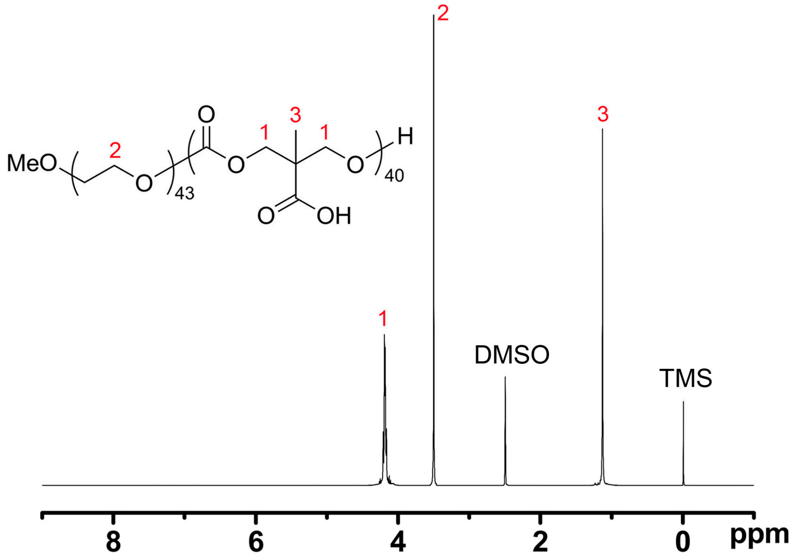

Figure S5. ^{1}H NMR spectra of mPEG$_{43}$-*b*-PMCC$_{40}$.

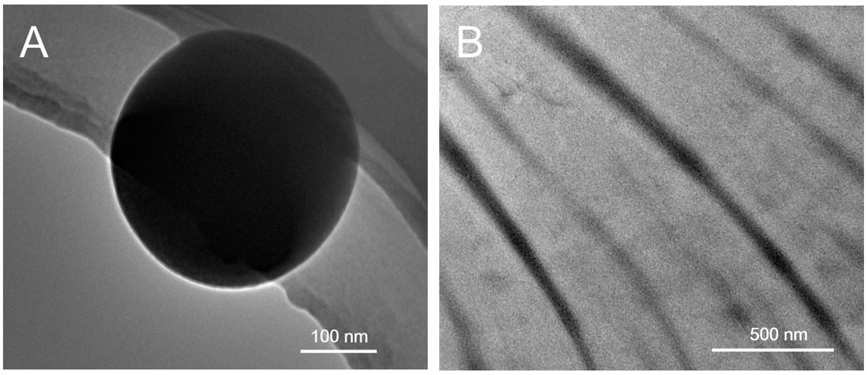

Figure S6. TEM images of $mPEG_{43}$-$PMCC_{25}$-$P(MCC\text{-}DHO)_{15}$ self-assembly obtained in water at (A) pH = 6 and (B) pH = 8.
